# Diagnostics of unmanned aerial vehicle with recurrence based approach of piezo-element voltage signals

**DOI:** 10.1038/s41598-024-68197-x

**Published:** 2024-07-26

**Authors:** Bartłomiej Ambrożkiewicz, Paweł Dzienis, Leszek Ambroziak, Andrzej Koszewnik, Arkadiusz Syta, Daniel Ołdziej, Vikram Pakrashi

**Affiliations:** 1 Faculty of Mechanical Engineering, Białystok University of Technology, Wiejska 45C, 15-351 Białystok, Poland; 2 Department of Technical Computer Science, Faculty of Mathematics and Technical Computer Science, Lublin University of Technology, Nadbystrzycka 38, 20-618 Lublin, Poland; 3 Centre for Mechanics, School of Mechanical and Materials Engineering, University College Dublin, Stillorgan Road, Belfield, Dublin 4, Republic of Ireland

**Keywords:** Piezoelectric sensor, Damage calibration, Unmanned aerial vehicle, Electric motor failure, Recurrence analysis, Mechanical vibrations, Engineering, Mechanical engineering

## Abstract

This work experimentally addresses damage calibration of an unmanned aerial vehicle in operational condition. A wide range of damage level and types are simulated and controlled by an electric motor via pulse width modulation in this regard. The measurement is carried out via established protocols of using a piezo-patch on one of the 8 arms, utilising the vibration sensitivity and flexibility of the arms, demonstrating repeatability of such protocol. Subsequently, recurrence analysis on the voltage time series data is performed for detection of damage. Quantifiers of damage extent are then created for the full range of damage conditions, including the extreme case of complete loss of power. Experimental baseline condition for no damage condition is also established in this regard. Both diagonal-line and vertical-line based indicators from recurrence analysis are sensitive to the quantitative estimates of damage levels and a statistical test of significance analysis confirms that it is possible to automate distinguishing the levels of damage. The damage quantifiers proposed in this paper are useful for rapid monitoring of unmanned aerial vehicle operations of connection.

## Introduction

Since their inception, Unmanned Aerial Vehicles (UAVs) have experienced a remarkable surge in utilization across various domains. Originally developed for military applications^1,2^, UAVs have rapidly expanded their scope to encompass a diverse range of industries, including entertainment, photography, product transportation, inspection and surveillance, agricultural applications, and even wireless communication networks^3–5^. Their widespread adoption in such a wide array of sectors highlights their versatility and potential.

The exponential growth and transformative impact of UAVs in recent years have turned them into a focal point of research and innovation. The rapid evolution of UAV technology, coupled with advancements in autonomous systems and artificial intelligence, has opened up new possibilities and applications for these unmanned platforms^6–9^. As a result, multiple disciplines are actively engaged in exploring the full potential of UAVs and finding novel ways to enhance their capabilities, safety, and reliability^10,11^. The ongoing developments in UAV technology and the exploration of their capabilities signify that these aerial vehicles are set to play an increasingly vital role in various industries and everyday life. As the scope of applications continues to expand, UAVs are poised to be transformative in multiple sectors and address challenges in innovative ways.

Amongst the various possibilities of development of UAVs, near real-time diagnosis of their operational conditions is important as UAVs are often becoming a strategic part of transportation, military and rescue missions^12,13^. Puchalski et al.^14^, investigates such diagnostics through the model-based detection derived on the identification of previously occurred states, and data-based detection through data recorded in real-time, respectively. The paper studies the operations of the physical system through, data-centric methods. A clear understanding of typical damages in unmanned aerial vehicles is required before choosing analysis methods and feature indicators for experimental datasets^15,16^. The methods can be broadly classified under two main groups. The first relates to actuator problems such as lock-in-phase, float, hardover or loss of effectiveness. The second is related to sensor problems like bias, drift, loss of accuracy, freezing and calibration error.

Diagnostics of drones have been typically conducted via a range of methods. These include time-series analysis searching for a defect, spectrum analysis, order analysis, modal analysis or time–frequency analysis^17^. Benini et al.^18^ proposed the diagnostics of actuator based on the acceleration measurements recorded with an inertial measurement unit (IMU). There, both time domain and frequency domain features were calculated for any given time and the specific dynamic state of UAV diagnosed with over 95% accuracy. Bondyra et al.^19,20^ proposed the diagnostics of a drone’s propelling system through the application of acoustic emission, and subsequently using the recorded signal to derive an effective neural network (NN) model. Banerjee et al.^21^ studied the defect of the ball bearing of the main rotor with FFT finding anomalies in drone operation. Al-Haddad et al.^22^ compared natural frequencies obtained from the computer aided model for the structure of the drone while experimentally demonstrating the advantage of data-based diagnostics. Based on experimental time-series data, Gośliński et al.^23^ conducted a model parameter estimation for a coaxial quadrotor. Another diagnostics method is the application of Attitude Heading Reference System (AHRS), which provide the information for drone, including pitch, roll and yaw^24,25^. AHRS is the extended alternative system providing the flight information in reference to Inertial Measurement Unit (IMU). Based on the above discussion, a data-driven approach for diagnostics of a UAV is commonly used and this continues to develop, as evidenced by a range of methods that are available for such analyses^26,27^.

Methods of analyses in this sector are recent and fast evolving. However, there is a need to establish calibration of damages in such UAVs using established detection methods and sensors via experiments. This paper addresses this need and expands on a recent work on sensor placement strategy and sensitivity of piezoelectric sensors for detecting damage in a UAV octocopter. One of the problems occurring in UAVs is the temporary shortage of energy in one or more actuators, causing a temporary loss of balance during flight. This paper investigates such damage experimentally in one of the drone's actuators and establishes damage quantification indicators using a piezoelectric measurement patch. In our previous research, we recently studied the best positioning of such piezoelectric patch for detecting such damage^28^. The conducted tests confirmed the sensitivity of the nonlinear recurrence analysis method for identifying damage within a narrow range (35%, 40%, and 50%). With such promising results, the next step is to establish repeatability of such results in the already investigated zone, extend the damage range and combination experimentally and to establish a damage quantification and calibration so that it can be used by others. This paper takes up all these requirements and presents an experimental damage quantification calibration for a wide range and combination of damages using such a piezo patch, via the established nonlinear recurrence indicator. The experiments consider extreme scenarios like complete power loss in one of the electric motors. More importantly, the experiments also establish the baseline values for damage extent quantification indicators for no damage scenarios, which are fundamental to comparing and using it for other situations.

Additionally, the paper improves the sensitivity and accuracy of the recurrence analysis exploiting correlations over longer time scales. The implementation method of the paper also now allows for a systematic differentiation of various operational states of the drive system based on Recurrence Quantification Analysis (RQA) indicators. Additionally, group statistical tests were conducted to confirm statistically significant differences in indicator values depending on the degree of damage. This continuation and extension of previous experiments presented in this paper for damage quantification thus allows for establishing repeatability of tests, expansion of experimental damage scenarios, calibration of damage extent indicators and a structured framework of application of the method proposed in^28^.

The remainder of this paper is as follows. In Section "Experimental setup and measurement system and methodology", information on the drone used for experiments, along with the measurement system are presented. Next, experimental scenarios are described in Section "Experimental design" along with details on specific damages. In Section "Data processing and recurrence-based methods", the applied method and time-series processing are presented for establishing damage extent indicators and their calibrations over the full range, along with baseline estimates and the extreme case of complete loss of power supply. Discussions on results obtained from conducted experiments is in Section "Results discussion", while Section "Conclusions" summarizes the paper showing the next steps of developing the proposed method of UAV diagnostics.

## Experimental setup and measurement system and methodology

### The idea of diagnostic motor by using piezo sensor

The propulsion system of the Vertical Take-Off and Landing (VTOL) UAV is composed with the Brushless Direct Current (BLDC) motors, propellers and the brushless motor Electronic Speed Controllers (ESC). These elements, apart from ESC can generate vibrations of the UAV arm also during their normal operational conditions. The amplitude and frequency of these vibrations can also depend on the flight phase as well as the internal conditions of the UAV system. Fractional resistance from dirty or worn-out bearings, damage propellers and engine falling out typically lead to change of vibration characteristics. To rapidly detect potential faults of these propulsion system elements, a piezoelectric patch is attached to the UAV arm so that it generates variable voltage depending on amplitudes and frequencies of UAVs arm deflections, as well as strains on the piezo patch. The variability of harvested voltage on the piezo patch can deduce the condition of faultless operation of the drive or the drive with impending or occurring damage so that appropriate action can be taken in the form of reconfiguration of the controls to ensure flight safety.

**Figure 1:**
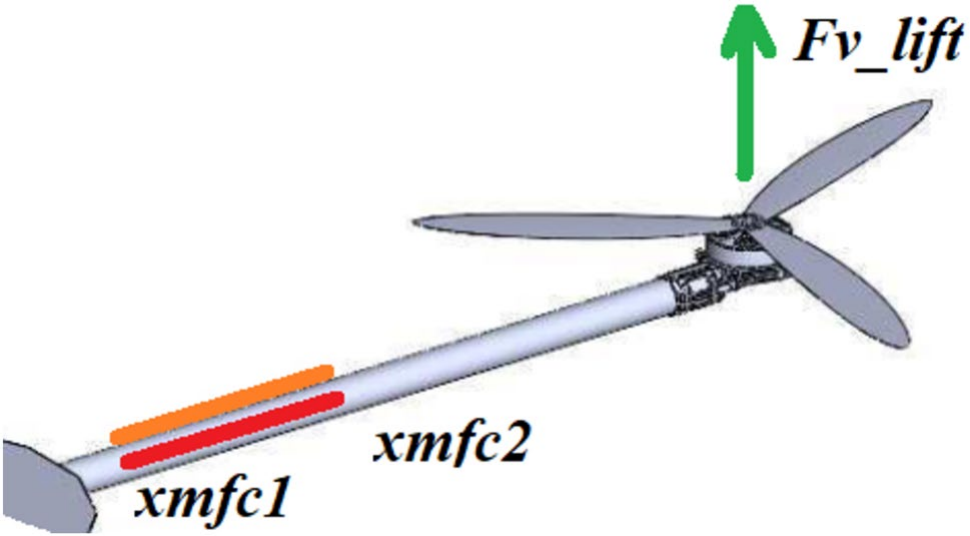
Conceptual model of UAVs arm with the integrated piezo-patch composites in two different orientations (on the top—orange line, on the side—red line).

Figure 1 presents a conceptual model of the UAV arm with an integrated piezo sensor, which is excited to vibration due to lifting force generated by the propulsion system on the free end of the arm. The general equation of transverse vibration of this beam structure with piezo patches located at position (*x*_*mfc1*_, *x*_*mfc2*_) and oriented in two different can be written as^29,30^:

1 $$ EI\frac{{\partial^{4} w(x,t)}}{{\partial x^{4} }} + m_{arm} \frac{{\partial^{2} w(x,t)}}{{\partial x^{2} }} - \rho_{MFC} t_{MFC} \frac{{\partial^{2} w(x,t)}}{{\partial t^{2} }} - \Gamma V(t)\left[ {H(x - x_{MFC\_2} ) - H(x - x_{MFC\_1} )} \right] = F_{v\_lift} \delta (x - x_{0} ) $$

where *E*—the Young modulus of the single arm of the octocopter, *I*—the inertia moment of the single arm of the octocopter, *m*_*arm*_—the mass per unit of length of the single arm, *w(x,t)*—the transverse vibration of the UAV arm at position *x* and time *t*, $\rho_{MFC}$—mass density of the piezo-patch composite, $t_{MFC}$—thickness of the piezo-patch composite, $\delta (x)$—Dirac delta function along the horizontal axis, *V(t)*—voltage flowing through the resistive load, $\Gamma$—electromechanical coupling factor of the piezo-patch sensor, *H*—the Heaviside function.

The current *i*(*t*) is associated with the strains of the piezo patch composite harvester and the electrical field applied to its electrode. The electrical circuit of this system can be written as^29^:

2 $$ C_{MFC} \frac{dV(t)}{{dt}} + \frac{V(t)}{R} + \Gamma \left[ {\int\limits_{{x_{mfc1} }}^{{x_{mfc2} }} {\frac{{\partial^{3} w(x,t)}}{{\partial x^{2} \partial t}}} dx} \right] = 0 $$

where *C*_*MFC*_—the capacitance of the piezo-composite sensor ($C_{MFC} = \frac{{\varepsilon_{33} w_{MFC} l_{MFC} }}{{t_{MFC} }}$) and *ε*_*3*3_—the strain of the piezo sensor, *w*_*MFC*_, *l*_*MFC,*_* t*_*MFC*_—the width, length and thickness of the MFC sensor, respectively, *R*—the resistive load.

The magnitude of the open-circuit voltage generated by this piezo-circuit for operation mode in the 31 direction excited by various states of the propulsion system can be expressed as

3 $$ V(t) = \frac{{E_{MFC} t_{MFC} d_{31} }}{{e_{33}^{\sigma } }}\varepsilon $$

where *E*_*MFC*_—the Young’s modulus of the piezo-patch composite, *d*_*31*_—the piezoelectric charge coefficient, $\varepsilon$—the strain on the piezo-patch element, $e_{33}^{\sigma }$—the dielectric permittivity of the piezoelectric material under a constant stress of σ.

### Experimental test

**Figure 2:**
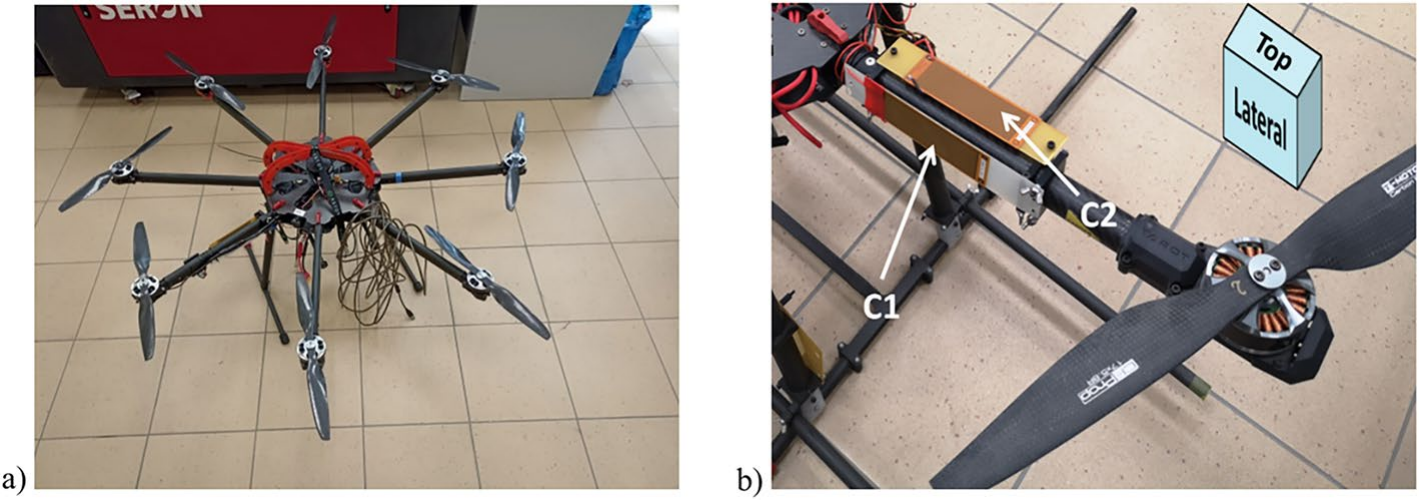
(**a**) Multirotor in eight-arm configuration, (**b**) piezo-patches located on one of drone’s arms (on the rectangular in top right corner there are specified planes considered in the experiment).

An unmanned multi-rotor helicopter type vehicle was considered for experiments. Due to the redundancy of the drives and the high thrust necessary for applications like precision farming or delivering relatively heavy payloads at the expense of flight time, an eight-arm configuration was chosen (Fig. 2a). The drone consists of carbon composites for the centerplate and aluminium for arms. The power source is a nominal battery of 6 lithium-polymer electric cells with a total voltage of 22.2 V. The drive units consists of a 15 × 5.5 propeller were coupled directly to the rotor of a BLDC motor (Tarot 5008). Eight drives ensures the stable operation of the structure. Each single drive unit is individually controlled by the Pixhawk Cube autopilot with a 50 Hz PWM signal through the Hobbywing 40A electronic speed controller (ESC). The mass of the octocopter ready for flight, i.e. with control and measurement equipment (200 g) and battery (1470 g) was 7125 g.

In Table 1, relevant octocopter properties are specified. For the analysis, two piezo-patches MFC 8528P2 were mounted on one of the arms of the drone at positions *x*_*mfc1*_ and *x*_*mfc2*_ (see Fig. 2b) and two different orientations on the side (C1) and on the top (C2), respectively. On the same arm, the change of the PWM signal powering-up the motor was initiated with 5 different levels. Methods of locating the piezo patches for advantageous signal responses are established in^28^.

**Table 1:** Characteristics of octocopter.

Parameter	Value
Distance between opposite motors	410 [mm]
Wheelbase diameter (top view)	1050 [mm] (motor axis to motor axis)
Maximum single-drive thrust	2.5 [kg]
Maximum take-off mass	13 [kg] (for stable flight)
Payload	6 [kg]
Power type and source	Lithium-polymer electric cells
Motor type	BLDC
Wind resistance	~ 16 [m/s]

**Figure 3:**
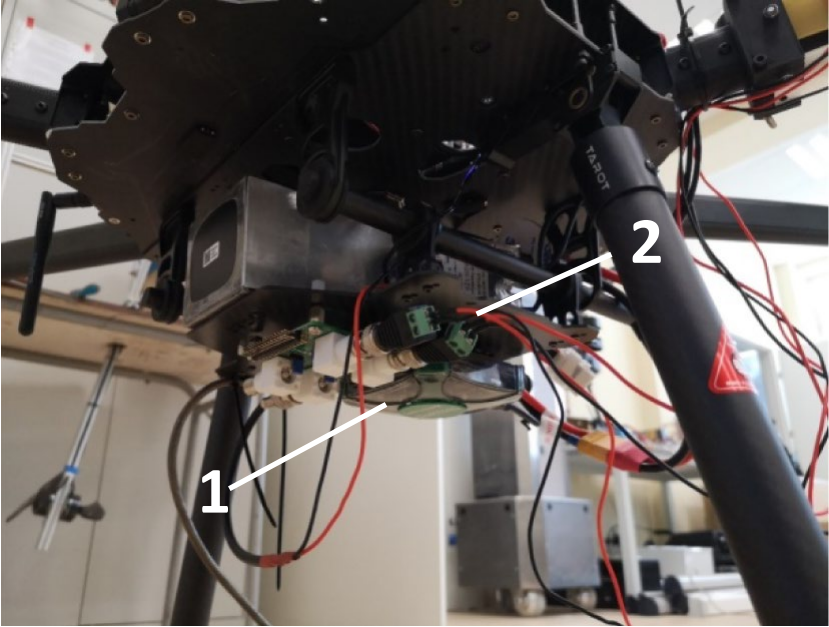
Implemented control system with data acquisition card digilent analog discovery 2 (1), SSR relay (2).

Digilent Analog Discovery 2 (Fig. 3) was used as a measurement card with the ability to measure two channels and generate two signal outputs, one of which (W1—wave generator no.1) was used to cause variable frequency damage. The implementation of the damage causes appearance of UAV arm vibrations and consequently, deformation of the piezoelectric elements located on arm in two following planes: on the lateral plane (C1—Fig. 2b) and the top plane (C2—Fig. 2b). The changes of voltage generated by piezoelectric elements were recorded by two available analog inputs (CH1, CH2) of Discovery 2 measurement card. The physical implementation of the damage in the Discovery 2 card generated a control signal for the Solid State Relay (SSR) switching on or disconnecting one of the three phases supplying the BLDC motor (Fig. 3). The same card was used to collect the experimental time-series data. The use of a solid state relay was necessary to obtain the appropriate switching frequency and to pass relatively high currents, even up to 20A.

**Figure 4:**
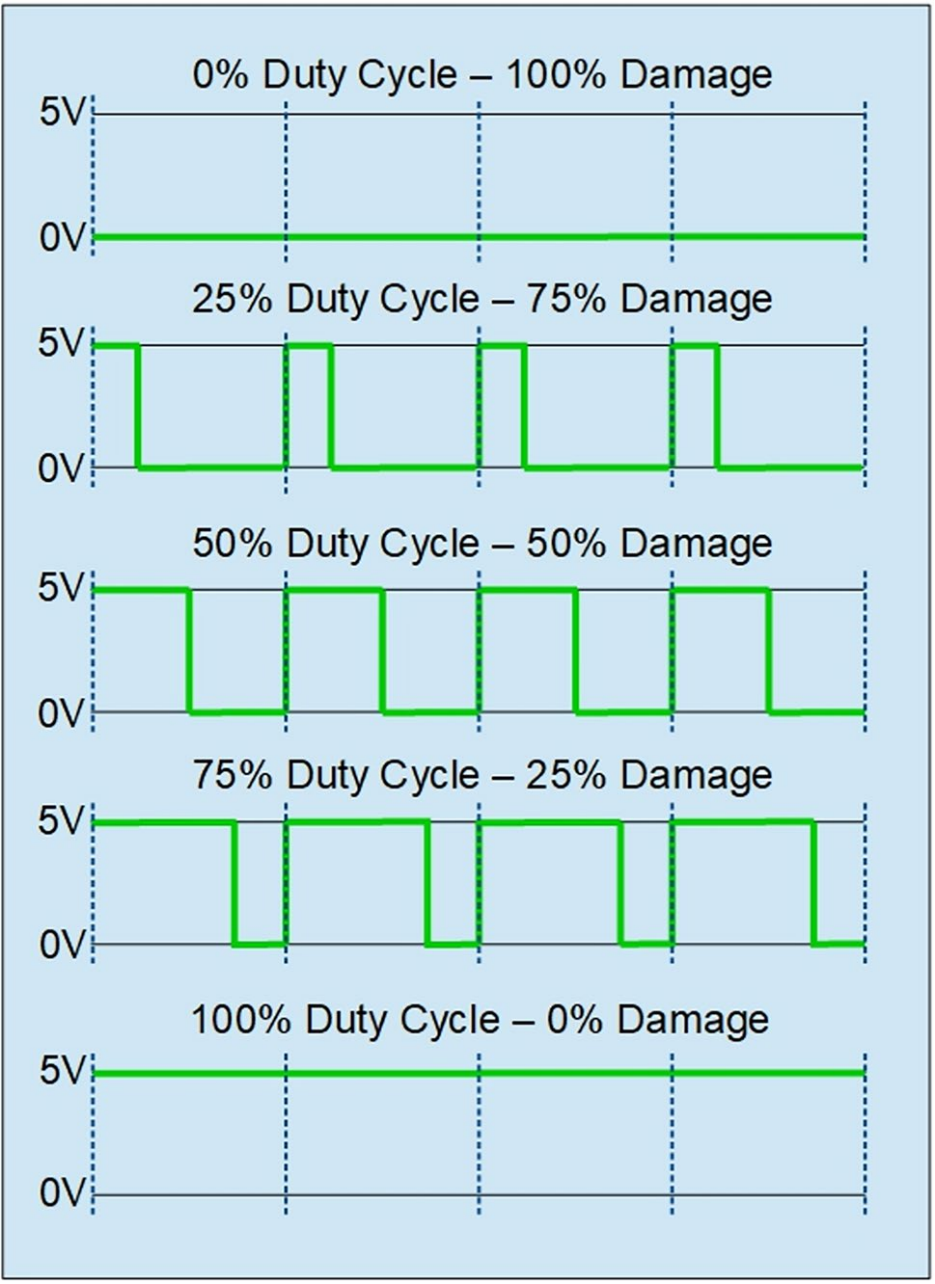
PWM frame for cases considered in the experiment.

An example of recorded time series of harvested voltage signals are presented in Fig. 4 and described in Section "Experimental design" in detail. Due to the occurrence of aperiodic disturbances in voltage time series, the signals were analysed using Recurrence Plots (RPs), which has been observed to be appropriate for detecting behavioural changes of these systems. Quantitative information of the damage conditions introduced experimentally were carried out by Recurrence Quantification Analysis (RQA).

## Experimental design

The most common issues encountered in the propulsion system of a multicopter are typically related to various components and subsystems. These potential problems can be specified following^31^:

- **Mechanical problems:** related to the damage of bearings, winding, unbalance, misalignment,
- **Electrical problems:** related to the damage of power management, battery pack, wiring overheating or quality of PCB and soldered joints,
- **Control problems:** related to the improper operation of ESC or processing of autopilot control signals.

Identifying and addressing above potential problems through regular maintenance, thorough inspections, and testing can help ensure the safety and reliable operation of the drone’s propulsion system. To enhance the reliability and safety of quadrotors, various methods have been employed, including control system reconfiguration and the use of fail-safe mechanisms such as parachutes or airbags. However, implementing these systems requires precise and fast detection of failures along with the time at which a failure occurs. One straightforward approach involves using an ESC with revolutions per minute (RPM) measurement for the motor^32^. These specialized ESCs are usually larger and heavier than the standard ones. Nevertheless, this method has drawbacks. While it can detect certain types of failures, it may not be capable of identifying all potential failures that could occur during a UAV flight. This RPM-based approach is effective in monitoring a drop in rotational speed, particularly in cases where the motor completely stops due to a power supply failure. However, it may not accurately detect other types of failures, such as partial propeller damage, bearing problems, or the loss of one or two power phases in the ESC. These situations may not result in a complete loss of rotational speed and could go undetected using this method. For more comprehensive fault detection and its isolation, the incipient winding fault detection in squirrel-cage induction motors is generating interest. This method utilizes fault characteristics and a robust observer to detect and isolate the source of the fault in the electric motor^33^. Such an approach is particularly valuable since a significant portion of UAV propulsion system failures involves situations where a complete loss of rotational speed does not occur. By employing sophisticated fault detection and isolation techniques like the one mentioned above, it becomes possible to identify a broader range of failure scenarios and respond accordingly. These advanced methods contribute to an overall increase in the reliability and safety of quadrotors, which is crucial for their successful deployment in various applications. Nevertheless, even though the above-mentioned methods show great accuracy in the detection of faults in electric motors, they can be hardly used in the prognosis of damage level, such the problem occurs in the increasing level of faults in rotational systems^33,35^. In particular the quantification of such damages over a wide range, along with experimental calibration of pristine condition is required but a clear experimental evidence base is missing. This paper addresses this and builds on detection capabilities observed with a short range of damages in^28^, while also expanding the damage conditions over a full range.

To achieve this, we consider tests for different levels of electric motor damage induced via the level of PWM signal sent to one of eight propelling motors. During the flight of the UAV this damage is initiated with the specific level of intensity and due to the damage, the drone starts to lose its stability. The loss of stability is the result of a temporary voltage shut-off on one of BLDC’s motor phases depending on the width of the PWM signal. After a while the electric motor starts to work properly. However, here we focus on the effect of the damage on flight stability. In Table 2, the considered level of damages over the full range is specified. To visualize, how PWM signal is sent to each phase of the propelling motor, Fig. 4 is presented. The percent of the PWM denotes the time by which the motor’s phase is shut-off leading to the interrupted operation.

**Table 2:** Level of damages considered in the experiment.

Damage	0%	25%	50%	75%	100%
Description	Correct operation	Width of PWM signal on one phase of 3-phase BLDC motor			

**Figure 5:**
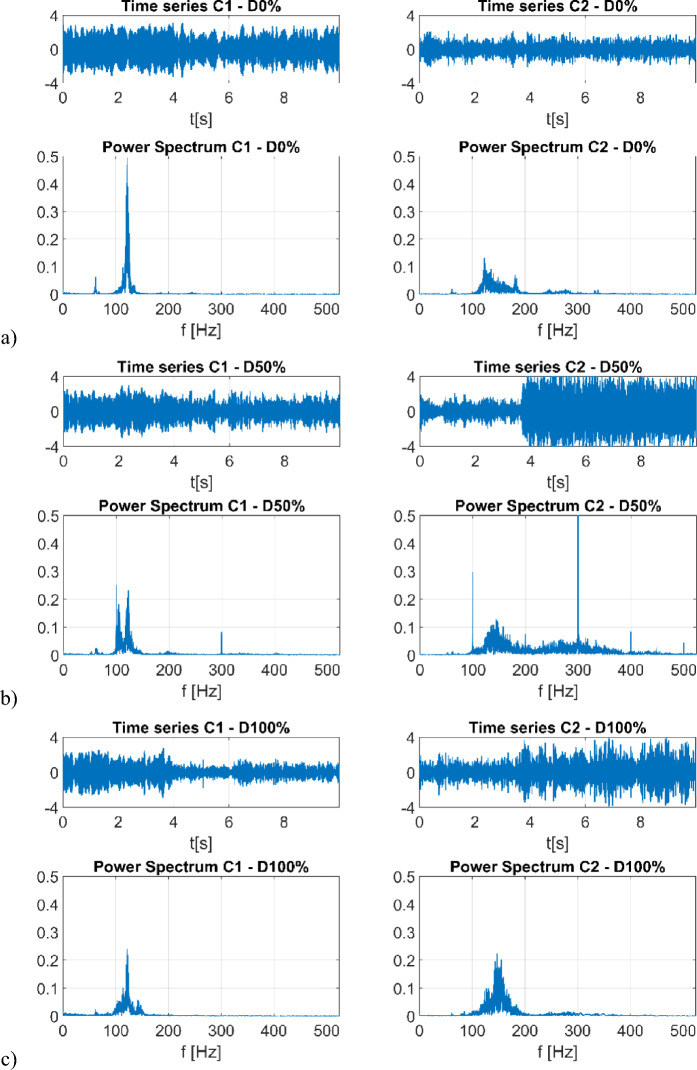
Voltage time-series and its FFT spectrum by specific level of damage (**a**) 0%, (**b**) 50%, (**c**) 100% for side sensor (C1—left panel) and top sensor (C2—right panel).

Five distinct scenarios of damage over a full range were meticulously examined, each characterized by variations in the duration of the PWM). This variation directly pertains to the specific impairment of one phase within the propelling motor. Throughout each case, the duration of the experiment was consistently set at 10 s. Notably, during the interval spanning from the 5th to the 6th second, one phase of the BLDC motor was intentionally deactivated. This deliberate action caused an immediate loss of stability in the drone’s operation. Consequently, the y-axis in Fig. 5, where the example time-series plots of voltage are presented, lacks specific voltage unit labels due to normalization. The sampling frequency for the experiments is 1048 Hz. Initially, identification of damage involved analyses of harvested open circuit voltage to via Fast Fourier Transform. Initial observation indicates that recorded time-series data exhibit comparable amplitudes, necessitating further exploration in the frequency domain. In Fig. 5, the power spectrum obtained from the analysis is presented. The power spectrum analysis of sample series with varying degrees of damage (D0%, D50%, and D100%) reveals different characteristics depending on the data source. In the case of signals recorded by sensor C1, a dominant component is clearly visible around 100 Hz, with a relatively narrow frequency band (from 100 to 140 Hz) in all cases. The occurrence of 50% damage (D50%) results in an additional component at 300 Hz, which disappears when the engine is completely shut down (D100%). A similar relationship can be observed in the power spectra for time-domain waveforms recorded by sensor C2. However, in this case, 50% damage (D50%) leads to a much broader frequency spectrum (from 100 Hz to nearly 400 Hz), including additional harmonics that are multiples of the dominant component (200 Hz, 300 Hz, 400 Hz, and even 500 Hz). To delve more deeper into the spectral characteristics as well as to create a robust damage quantification marker, it nonlinear recurrence analysis is carried out, which can exploit underlying patterns and complexities in the data that may not be apparent solely through methods like Fourier transform.

## Data processing and recurrence-based methods

The complexity of dynamics in drones has led to the application of various methods, which can be categorized into four main types: signal processing, statistical analysis, pattern recognition, and information fusion^31^. When dealing with time series derived from the experiments, it is common for the data to possess a non-stationary nature, necessitating multi-stage treatment such as filtering, normalization, or decomposition to achieve clear and satisfactory results^36^. However, determining the characteristic frequency of the drone during the flight cannot be adequately described with the empirical formula, due to its complexity. Moreover, numerous additional frequency peaks appear in the time series spectra, so alternative and advanced data treatment has to be applied. Here, the raw voltage time series were taken into account. A promising method for examining short-time series data is recurrence analysis^37,38^ which has been established to be useful for this type of system and damage^27^ via its sensitivity to detecting the system’s return to the same state after a certain period but has not been explored for quantification of damages or for a full range of damage either. The word damage can be understood as a disturbance in the normal operation of the octocopter drive system. Recurrence Plots (RPs) also provide qualitative information about the system’s non-stationarity, periodicity, pitfalls, or signal-to-noise ratio^39^. For a more efficient analysis of the system’s dynamics and to obtain quantitative information, Recurrence Quantification Analysis (RQA) is preferred^40,41^.

### Recurrence plots (RPs)

Recurrence plots (RPs) can qualitatively analyze the periodicity of dynamical systems, revealing instances when phase space trajectory of a system returns to roughly the same area in the phase space at different times. The method involves constructing a two-dimensional square matrix, typically denoted as *R*, where the element *R*_*i,j*_ indicates whether there is a similarity between the states of the system at time instant *i* and time instant *j*. In *R*_*i,j*_, a value of 1 represents a recurrence point, meaning that the system's state at time instant *i* is close enough to the state at time instant *j* to be considered a recurrence. On the other hand, a value of 0 indicates no recurrence, implying that the states at time instants *i* and* j* are significantly different. The process of creating recurrence plots involves calculating a distance measure (e.g., Euclidean distance) between the states of the system at various time points and applying a threshold to determine whether a recurrence is present (marked as 1) or not (marked as 0). Recurrence plots offer valuable insights into the underlying dynamics and can help identify changes in periodicity of nonlinear systems^42,43^. The *R* matrix is defined as:

4 $$\left[{R}_{i,j}\right]=\Theta \left(\varepsilon -\Vert \left\{{x}_{i}\right\}-\left\{{x}_{j}\right\}\Vert \right), i,j,\dots , N$$

where, *N* is the number of considered states, $\left\{{x}_{i}\right\}$ and $\left\{{x}_{j}\right\}$ are vector system states at times $i$ and $j$, respectively, $\varepsilon$ is a threshold distance, $\Vert \Vert$ indicates a norm (usually the Euclidean norm) and $\Theta$ is the Heaviside function.

The distance matrix obtained consists of zeros and ones forming the recurrence plot (RP):

5 $$\left[{R}_{i,j}\right]=\left\{\begin{array}{c}1:\left\{{x}_{i}\right\}\approx \left\{{x}_{j}\right\},\\ 0: \left\{{x}_{i}\right\}\ne \left\{{x}_{j}\right\},\end{array}\right.i,j,\dots , N$$

where $\left\{{x}_{i}\right\}\approx \left\{{x}_{j}\right\}$ are the points from the ehaviourood of radius *ε* (defined according to the applied norm—Euclidean in our case).

The values of $\left[{R}_{i,j}\right]$ plotted using black points for the value 1 and white points for the value 0 create the RP plot that gives qualitative information about the dynamics of the system via certain patterns, for example:

- **Isolated points:** Isolated points indicate regions of the phase space where the system exhibits noise or stochastic ehaviour. These points suggest that the system’s trajectory is visiting seemingly unrelated regions in the phase space over time.
- **Diagonal lines:** Diagonal lines are often associated with periodic or quasi-periodic ehaviour in the system. Such lines suggest that the system’s trajectory returns to similar regions in the phase space at regular intervals, indicating the presence of periodic vibrations or oscillations.
- **Vertical lines:** Vertical lines can be indicative of laminar or stable states in the system. These lines suggest that the system remains relatively unchanged over time, possibly settling into a steady-state or equilibrium configuration.

### Recurrence quantification analysis (RQA)

The quantitative approach for recurrence analysis was initially proposed by Trulla et al.^44^ and extensively discussed in works by Zbilut et al.^45^. The use of Recurrence Quantification Analysis (RQA), consists of a set of statistical indicators based on patterns in RPs^46^. Individual indicators determined based on the statistics of diagonal or vertical lines are presented for completeness:

#### Quantifiars based on diagonal lines (related to predictability and stability of the system)

Determinism *DET*—the proportion of recurrence points forming diagonal lines relative to all the recurrence points, corresponding to predictability of the system:

6 $$DET=\frac{\sum_{l={l}_{min}}^{N}lP(l)}{\sum_{l=1}^{N}lP(l)}$$

where *P*(*l*) is the histogram of the diagonal line, *N*—length of a data series, *l*_*min*_—predefined minimal length of a diagonal line,

Average line length of the diagonal lines *L*—the average time that two states of the system are similar, segments of the phase space trajectory stays come close to each other, corresponding to the characteristic timescales:

7 $$L=\frac{\sum_{l={l}_{min}}^{N}lP(l)}{\sum_{l={l}_{min}}^{N}P(l)}$$

Length of the longest diagonal line *L*_*max*_—the longest time that two states of the system are similar:

8 $${L}_{max}=\text{max}\left(\left\{{{l}_{i}}_{i=1}^{{N}_{l}}\right\}\right)$$

where ${N}_{l}={\sum }_{l\ge {l}_{min}}P(l)$ corresponds to the total number of diagonal lines in the matrix.

Entropy *ENTR*—the entropy of diagonal lines, corresponding to complexity and randomness of the system:

9 $$ENTR=-\sum_{l={l}_{min}}^{N}p\left(l\right)ln p(l)$$

where: *p*(*l*)—the probability of finding a line of with exactly *l* length.

#### Quantifiars based on the vertical lines (related to intermittency and stability of the system)

Laminarity *LAM*—the proportion of recurrence points forming vertical lines relative to all the recurrence points, corresponding to stability of the system:

10 $$LAM=\frac{\sum_{v={v}_{min}}^{N}vP(v)}{\sum_{v=1}^{N}vP(v)}$$

where *P*(*v*) is histogram the vertical line, *N*—length of a data series, *v*_*min*_—predefined minimal length of a vertical line.

Average line length of the vertical lines *V*—the average time that states of the system in trapped in some state, corresponding to intermittency of the system:

11 $$V=\frac{\sum_{v={v}_{min}}^{N}vP(v)}{\sum_{v={v}_{min}}^{N}P(v)}$$

where *N*_*v*_—is the absolute number of vertical lines.

Maximal length of the vertical lines *V*_*max*_—the longest time that the system stays in particular state:

12 $${V}_{max}=\text{max}\left(\left\{{{v}_{l}}_{l=1}^{{N}_{v}}\right\}\right)$$

where *N*_*v*_—is the absolute number of vertical lines.

## Summary of experimental procedure and data analysis

**Figure 6:**
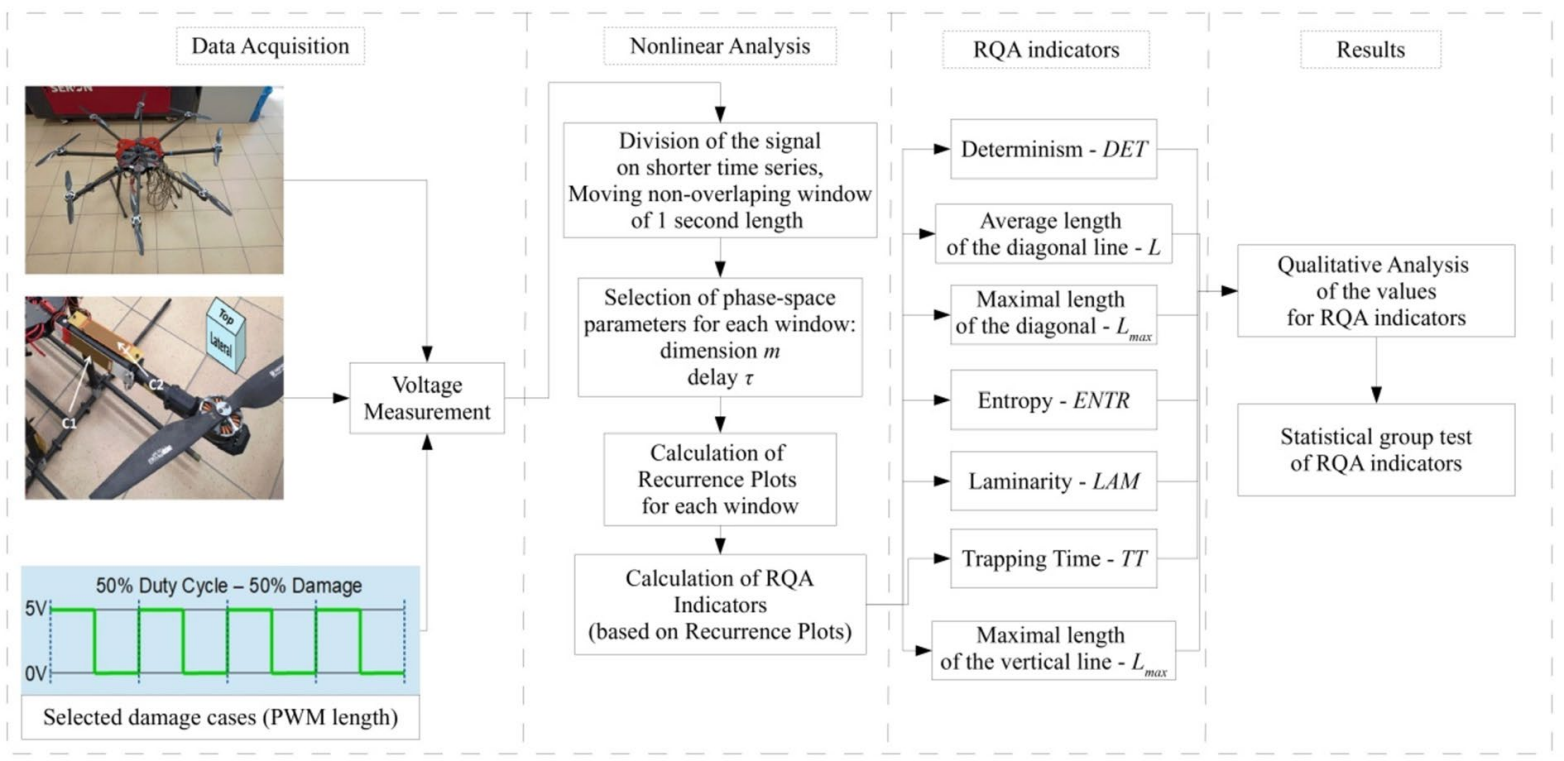
Flowchart for the experimental procedure, data analysis, and results.

In order to present the experimental procedure and the data analysis in a better manner, the flowchart of the whole procedure is presented in Fig. 6. Starting from the left, the octocopter used in the experiment is presented, while at the other photos at this panel the electric BLDC motor is shown in which the damage is induced. At the same panel, the piezoelectric sensors are depicted that are used as the sensors for the vibration analysis of the structure. Referring to Fig. 5, the experimental time-series are obtained that are further used in the nonlinear analysis. At this time instant, the obtained voltage time-series are divided into short-time segments, one second each, and based on them the recurrence indicators are calculated. At the final stage, the qualitative analysis of obtained recurrence indicators is performed supported with the statistical group test.

## Results discussion

The recurrence method requires setting the values of certain parameters which has to be aligned to the system and the detection requirements Comparison of the distances between the states of the system takes place in the space of all states, which is usually not possible in the case of experimental systems where one comparison of the distances between the states of the system takes place in the space of all states. Under such circumstances, one can reasonably reconstruct the space of all states using by choosing the time delay $\tau$ and the embedding dimension $m$ in the analysis^47^. In order to obtain a topologically equivalent state space which, amongst other things, preserves distances. The time delay is responsible for correlating the individual components of the state vector and an appropriate choice of this value maintains correlations between data on short and long time scales. Typically, this value is determined as the first minimum of the Mutual Information Function^48^. The value of the embedding dimension should be large enough to maintain the distance between nearby states of the system. In this case, the False Nearest Neighbors Fraction function is usually assumed to be zero^49^. The embedding parameters were determined independently for each measurement point, in this case for the degree of motor damage. In all cases, the time delay value was $\tau =2$, and based on this, the calculated embedding dimension value was $m=3$. The independence of embedding parameters indicates, on one hand, a low level of noise (small values of $\tau$ and $m$) and the repeatability of the experimental procedure, establishing the effectiveness of positioning indicated in^28^ as well. On the other hand, the dimension of state space remains unchanged with varying degrees of damage, which suggests that the damage can be interpreted as a change in the initial conditions of a nonlinear experimental dynamic system. The chosen method of recurrence analysis allows for the identification of such changes. Under such circumstances, establishing baseline conditions for damage quantification becomes particularly important.

**Figure 7:**
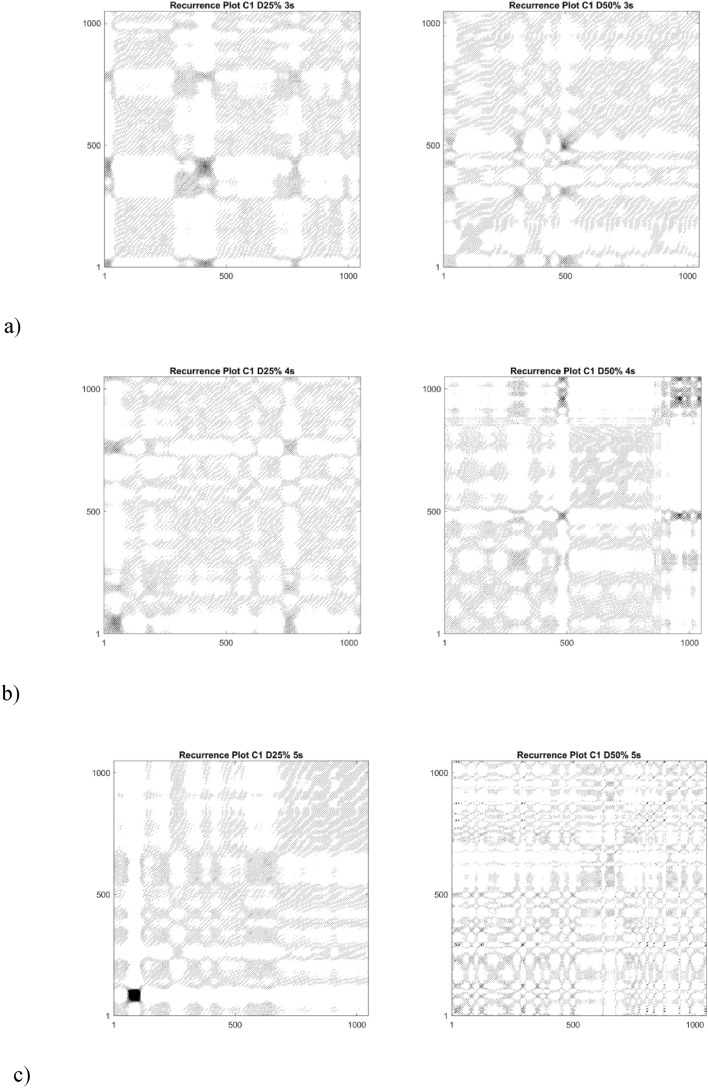
Comparison of recurrence plots for two different level of damages, i.e. 25% (left panel) and 50% (right panel) by following time (seconds) of the test (**a**) 3 s, (**b**) 4 s, (**c**) 5 s.

Selected recurrence plots generated on the basis of experimental data recorded by the C1 sensor with a length of 1 s for two different PWM signal values, i.e. 25% and 50%, are presented in Fig. 7. All analyses were conducted using Matlab software^50^, and the recurrence analyses were performed using an external extension for Matlab^51^.

Obtained RP plots (Fig. 7) for different PWM signals qualitatively differ, and based on their structure it is possible to distinguish the moment of switching off one of the motor’s phases between the 4th and 5th second. More precisely, they are characterized by longer diagonal lines (Fig. 7a), which indicates greater repeatability of the drive system. At second 4 (Fig. 7b), it can be seen that the diagonal lines become shorter and the uniformity of the RP plots decreases. The shortest diagonal lines appear in second 5 (Fig. 7c), which contains a record of the operation of the damaged motor. Vertical structures and empty white zones indicating a loss of correlation that were not present before (Fig. 7a,b) also clearly appear. The detection of transient states with RP is possible, but it is worth cross-checking it with the quantitative method. Here, 7 recurrence quantifiers (based on diagonal and vertical lines) were calculated in the whole range of tests (C1 and C2 sensors) and for all damage cases ranging fully from 0 to 100%, as presented in Fig. 6.

**Figure 8:**
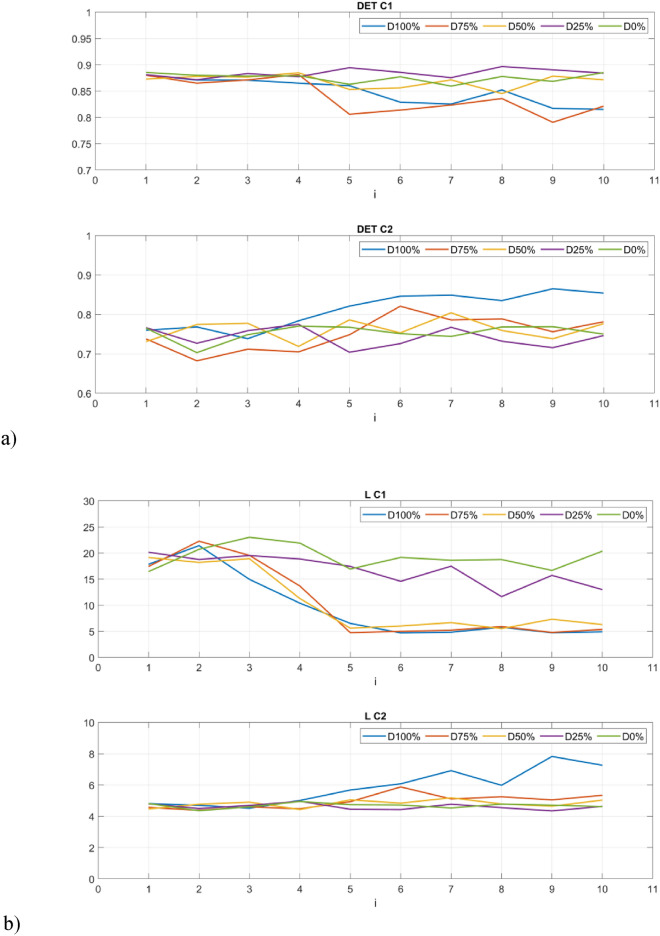

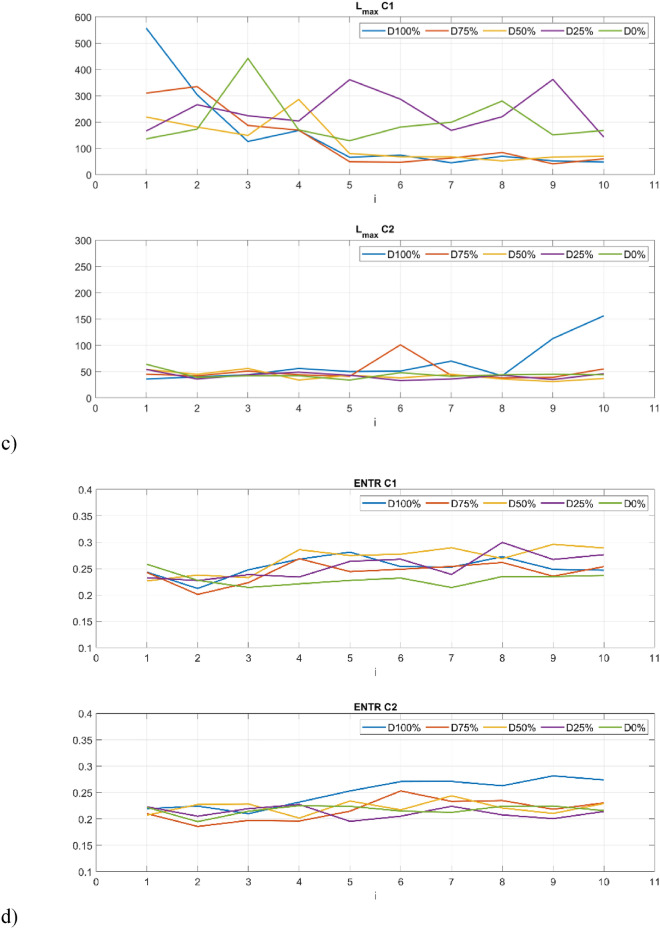
Results of RQA indicators based on statistics of diagonal lines.

The first group of studied recurrence quantifiers are those based on the diagonal lines in recurrence plots (Fig. 8). The first studied quantifier is determinism (DET) which refers to the system’s predictability. Determinism is observed to be sensitive to the change in the drone’s operation. For example, in the 5th second of the flight, there is an observable decrease of determinism, This is particularly pronounced for C1 sensor and highest damages, as one might expect: (100% and 75%—Fig. 7a). This is also aligned with the obtained recurrence plots (Fig. 7). During the undamaged operational conditions, determinism is on a constant level and this level is required to be determined experimentally for adaptation of the approach to other UAVs.

The average length of the diagonal line (L) provides comparable insights into the system’s dynamics as determinism, as they closely track each other’s trends. However, the quantifiers considered in the paper serves as a more reliable indicator of the stability of periodic intervals, offering an authentic representation within the RP. Qualitative changes of the quantifiers considered during the disturbance in flight is clear and there is a significant decrease (up to 5 times) in the length of the diagonal line (Fig. 8b) for higher damage (50–100%) for vibrations harvested by sensor C1.

The maximal length of the diagonal line (L_max_) in contrast to the average length of the diagonal line highlights the moment of dynamic disturbance in a better manner. In the 5th second, the radical decrease of the quantifier’s value is observable. Such behavior is especially visible by a value of damage higher than 25%.

Another quantifier based on statistical analysis of diagonal lines is considered namely entropy (ENTR), which refers to information about the disorder of oblique line lengths. Greater variation in line lengths is characterized by higher entropy, while smaller variation indicates lower entropy. A slight increase in entropy can be observed at the moment of damage occurrence, while during its duration, it fluctuates slightly. Here, entropy is distinguished with the motor completely turned off (D100%), which achieves the highest values, but only in the case of data from sensor C2.

**Figure 9:**
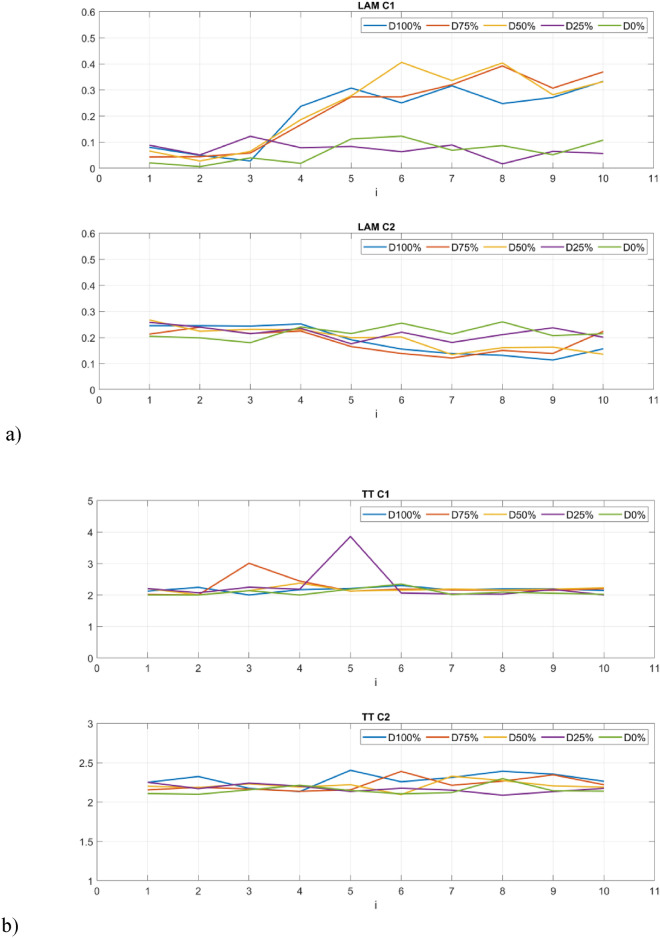

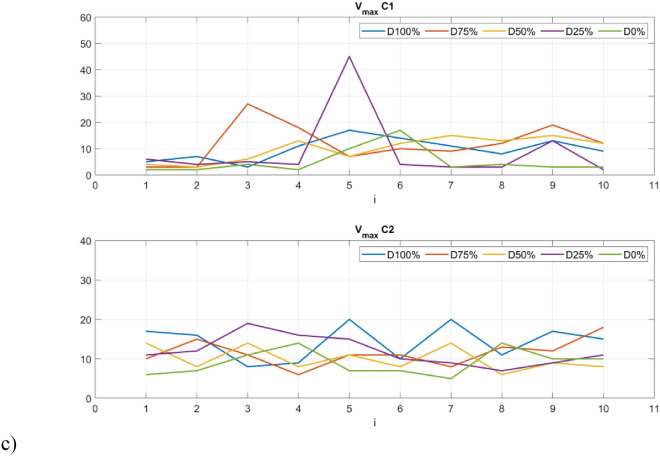
Results of RQA indicators based on statistics of vertical lines.

The second group of quantifiers is based on vertical lines created with the aggregated recurrence points in the RP. The first quantifier is the laminarity (LAM) which reflects the time in which the system is trapped. The mentioned quantifier is the opposite of determinism and is highly sensitive to the change of the PWM signal. The level of laminarity increases for damage higher than 25% (Fig. 9a) especially for the C1 sensor and stays at the same level for lower damage and proper motor operation.

Trapping time (TT) in this context denotes the mean duration of the vertical lines within the measurement time scale, representing minor fluctuations in dynamical responses. The variations in trapping time align with alterations observed in recurrence plots, with its upward trajectory correlating to the level of the PWM signal.

The last of the considered quantifiers is the maximal length of the vertical line (V_max_) that exhibits close similarities with trapping time, as square-shaped patterns give rise to progressively elongated vertical lines as the damage increases. This metric closely mirrors the observed structures within recurrence plots.

The values of the RQA indices presented in Figs. 8 and 9 illustrate their change in 1-s intervals depending on the value of the set damage. You can see that the time between 4 and 5 s when the damage is triggered is crucial here. Further analyses were limited to the final 5 s, discarding the first 5 s. The statistical significance between the groups of RQA indicators constructed in this way was tested using the Kruskal Wallis test. This is a non-parametric hypothesis test that compares three or more (five in our case) independent groups. It is used to determine whether at least one of the groups has a different median than the others. It is the non-parametric equivalent of the one-way ANOVA and is typically used when normality assumption is violated. At a typical significance level of 0.05, the null hypothesis H0 was formulatedas: The median of RQA indicators in 5 groups is equal, and the alternative hypothesis H1: The median of at least one RQA indicator differs from the others. The p-values for the Kruskal Wallis test conducted using Matlab software are summarized in Table 3.

**Table 3:** The p-values for Kruskal–Wallis test.

RQA index	DET		L		L_max_		ENTR	
Sensor	C1	C2	C1	C2	C1	C2	C1	C2
Diagonal-line based quantificators								
p-value	0.00009	0.00038	0.00007	0.00005	0.00017	0.0263	0.00035	0.00050

RQA index	LAM		TT		V_max_			
Sensor	C1	C2	C1	C2	C1	C2		
Vertical-line based quantificators								
p-value	0.00014	0.0023	0.00007	0.0030	0.1960	0.0321		

**Figure 10:**
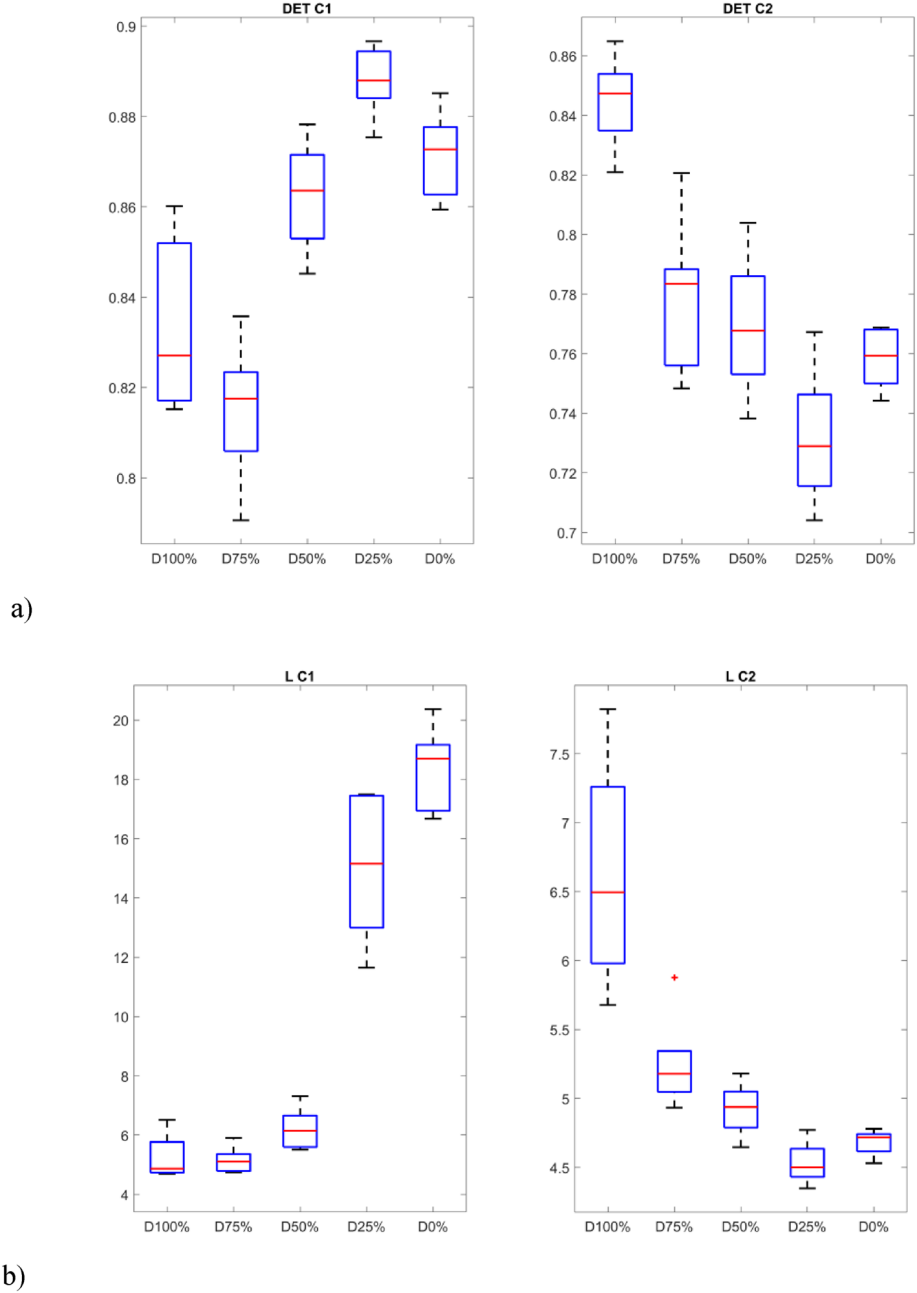

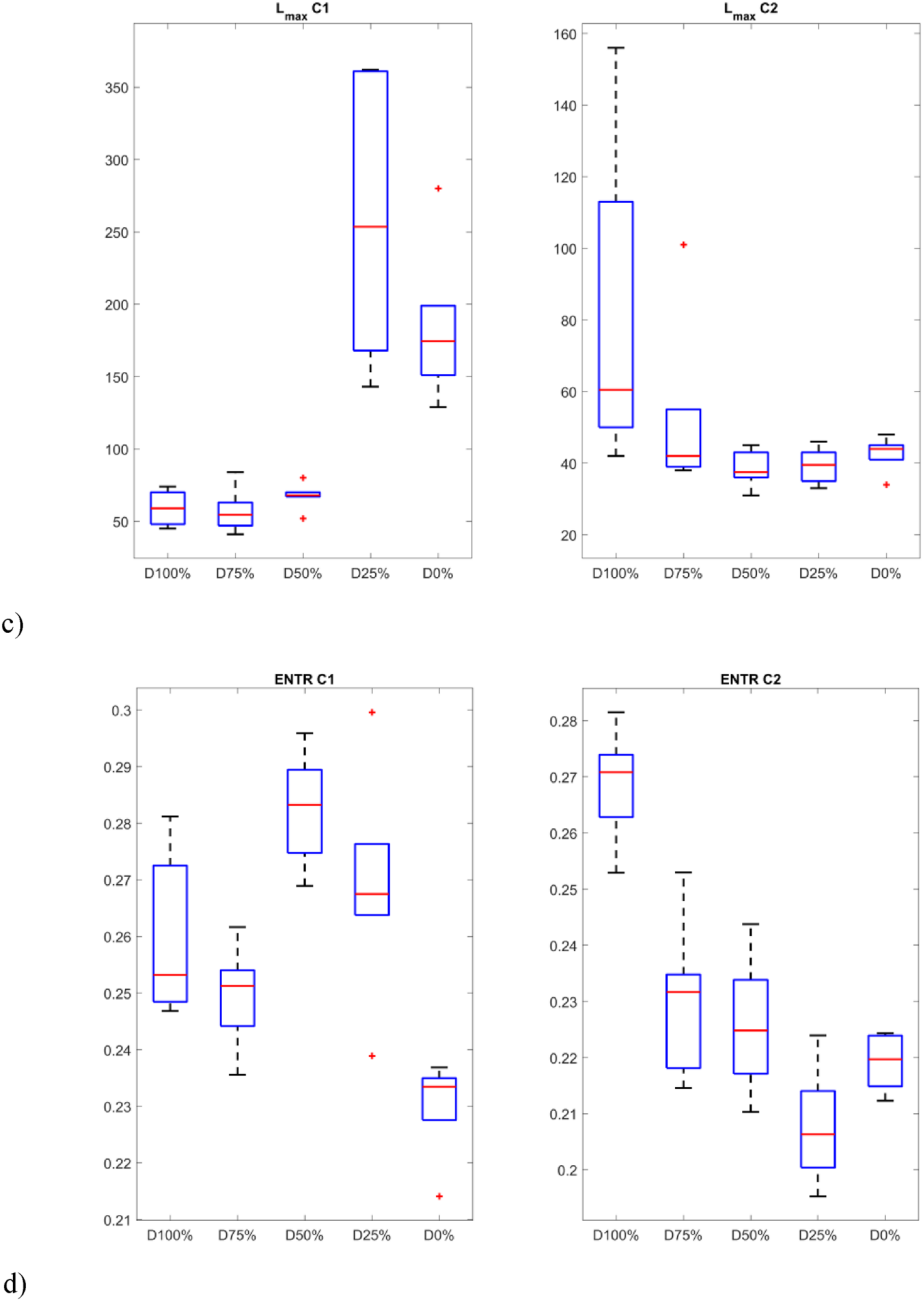
Box plots of diagonal line based quantificators.

**Figure 11:**
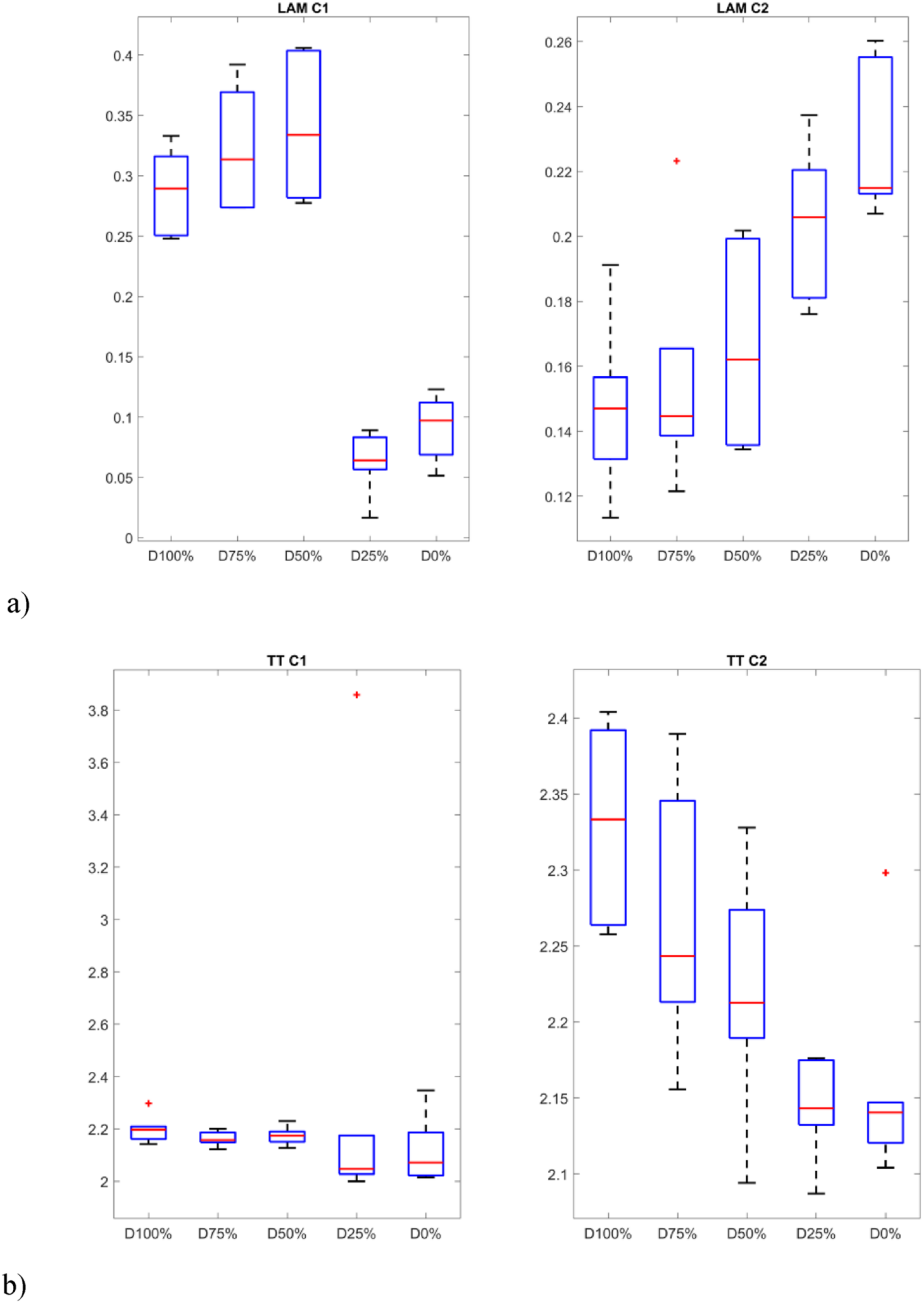

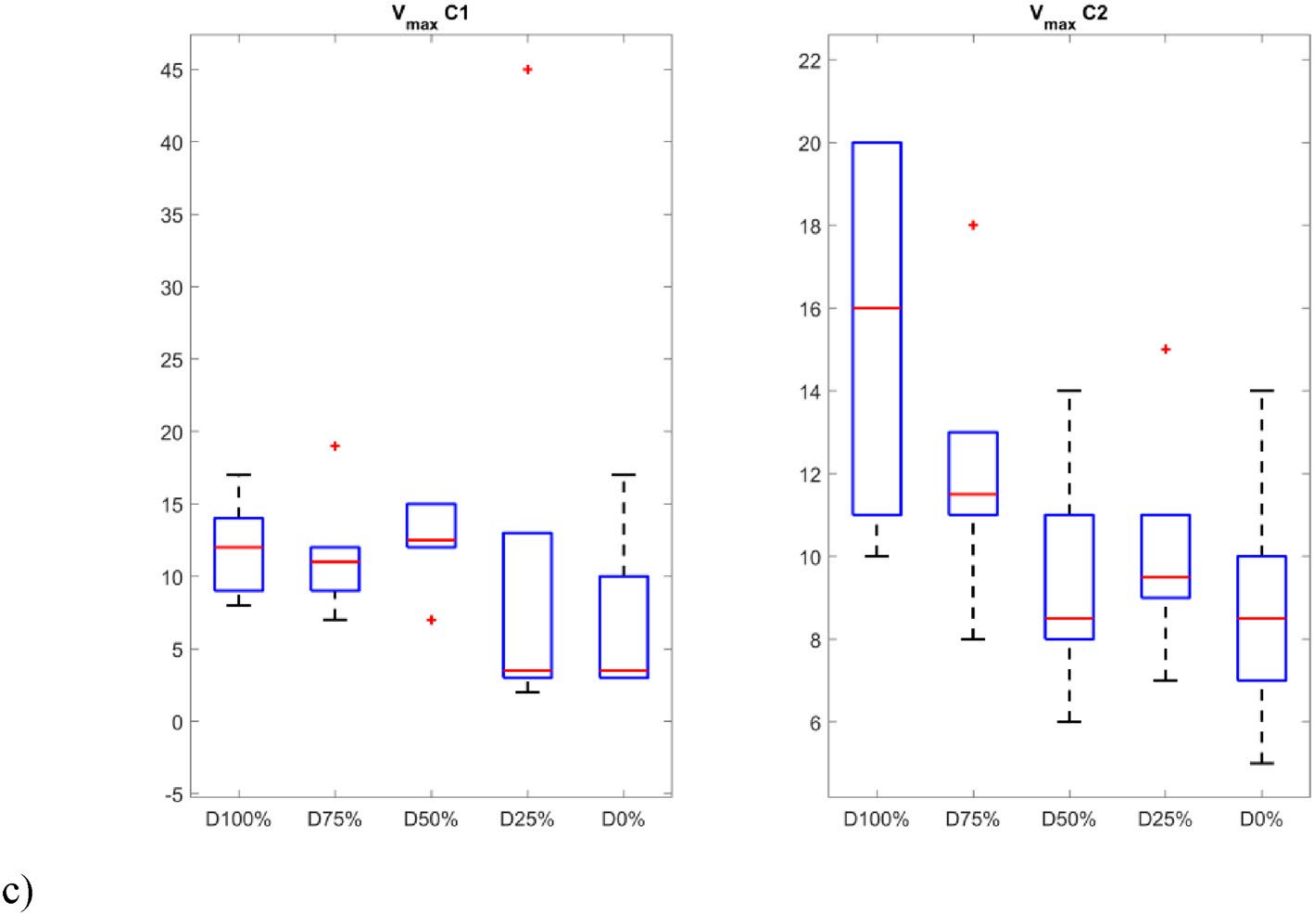
Box plots of vertical line based quantificators.

The p-values show that the samples differ significantly from each other (p-values < 0.05) in almost all groups, except for the V_max_ index. In addition, the smallest values occur for indicators calculated based on signals obtained from the sensor C1. The distinction between the individual groups (damage severity) can be presented graphically using box plots (Figs. 10 and 11). Consequently, it is possible to automate the detection of damage and also distinguish between different levels over a full range. In fact it can also provide indicators for creating system analyses or control algorithm changes for such UAVs^52^.

The statistics presented in the box plots also show significant differences between the values of the RQA indexes and the damage value most visible for the data obtained from the C1 sensor (discussion of which is limited). In particular, it should be noted the change in the periodicity of the system in the values of the indices calculated on the basis of diagonal lines, i.e. DET, L_max_, L, and ENTR. In the case of Determinism (DET), it is highest for no damage (D0%) and least damage (D25%), and then decreases with increasing damage (D50% and D75%) to finally increase slightly in the case of a switched off motor (D100%) with highest spread. A similar variation occurs in the statistics of the L_max_ indicator, where the largest values occur for minimal damage (D25%) and no damage (D0%), but with the largest range in the case of the former. However, they are still several times higher than in the other cases (D50%, D75% and D100%). Exactly the same relationship is seen in the L quantifier statistics. The diagonal line entropy index (ENTR) shows some differences (lowest for D0%), but no clear gradation. In the case of indicators based on statistics of vertical lines, laminarity (LAM) shows the most significant difference, where the values for no damage (D0%) and least damage (D25%) are the lowest, while in the other cases, they increase (the highest for D50%).

## Conclusions

This article reports the study on the dynamics of an 8-arm drone (octocopter) in terms of disturbed operations or damage induced by switching off one of the motor’s phases and creating scenarios that cover a wide range of such damage, including undamaged baseline conditions. The difference between the damage scenarios were maintained by input PWM signal to the motor’s phases, with a total of five different levels of damage considered (from 0 to 100% in 25% steps). Piezo-sensors mounted on one of the drone’s arms recorded open circuit voltage via vibration energy harvesting during 10-s flights for each damage conditions and detection of damage was carried our via recurrence analysis, which established the repeatability of the approach, while extending the experimental evidence base to a full range of damage.

Obtained time series data of the harvested voltage or a Fourier transform of it did not allow for a clear distinction between the levels of damage, but in some cases, they did allow for identifying the time of damage occurrence (Fig. 5). Recurrence analysis successfully distinguished between the different damage levels qualitatively via recurrence plots. Quantitative indicators for damage extent calibration were developed through recurrence indicators for future adaptation and use, along with calibration of undamaged reference which is required for different UAVs. While recurrence plots allowed for visualising changes in damage over the full the RQA indicator values obtained in this paper establishes damage quantification. The most prominent indicator in this regard was based on diagonal line statistics of recurrence plots. Test of significance performed on the different levels of damage indicates that it is possible to use the proposed quantifiers for automatic detection of changes in damage levels via a statistical quality control type approach. The most relevant sensor location in terms of information about the state of the system was that mounted on the side arm of the UAV.

A key benefit of the results lie in establishing the damage quantifier values for maximum exploitation of sensor placement strategies and recurrence approaches which has recently shown promise for such systems. This approach allows for checking of throttle levels in non-destructive way, as well as different levels of PWM signal for both indoor and outdoor tests, where the UAV was connected with a cable. A drawback of this approach is the lack of a uniform adhesive layer between the piezo-sensor and the frame of the UAV structure, which can affect the voltage generated by each piezo element located on the frame of the UAV and also change their as-instrumented characteristics.

There are several directions that can be taken from this step, which augments the initial evidence in^28^ to quantification and exploration of the full range of damage. Application of artificial intelligence methods to the obtained quantifiers by the different levels of PWM signal will be particularly relevant for the automation that has been now shown to be possible through test of significance leading to recognition of damage levels AA Joint Recurrence Plot^53^ can be considered to examine the correlation between the data obtained from two piezo-patches. Comparison of extensive sets of features^54,55^ can also be relevant for aerospace components^56^, as well as use of new types of sensors, including biopiezoelectrics^57^ and novel techniques like neuromorphic computing frameworks^58^.

This approach can be very useful for specialized UAVs in which on-board equipment (sensors, optical equipment, payload) is often several times higher in terms of weight or cost than the flying platform itself. This method can be used not only for multi-rotor UAVs but also for other types of aircraft such as airplanes or VTOL (Vertical Take-Off and Landing) aircrafts and the damage calibration over a full range as demonstrated in this paper has to be done via similar experiments, including the establishment of undamaged baseline.

## Data Availability

The datasets used during the current study available from the corresponding author on reasonable request.
